# Beware the respiratory epithelial adenomatoid hamartoma—a malignant masquerador

**DOI:** 10.1093/jscr/rjab007

**Published:** 2021-02-06

**Authors:** Shivani Angelique Kumar, Connor O’Meara, Sue Fredericks, Thomas Havas

**Affiliations:** Prince of Wales Hospital Otolaryngology Head and Neck Research Group, Sydney, New South Wales, Australia; Department of Otolaryngology, Prince of Wales Hospital, Sydney, New South Wales Australia; Faculty of Medicine, University of New South Wales, Sydney, New South Wales, Australia; Prince of Wales Hospital Otolaryngology Head and Neck Research Group, Sydney, New South Wales, Australia; Department of Otolaryngology, Prince of Wales Hospital, Sydney, New South Wales Australia; Histopath Diagnostic Specialists, Sydney, New South Wales, Australia; Prince of Wales Hospital Otolaryngology Head and Neck Research Group, Sydney, New South Wales, Australia; Department of Otolaryngology, Prince of Wales Hospital, Sydney, New South Wales Australia; Faculty of Medicine, University of New South Wales, Sydney, New South Wales, Australia

## Abstract

Respiratory epithelial adenomatoid hamartoma (REAH) is a rare benign tumour, which can masquerade as a sinonasal malignancy. Commonly arising from the posterior nasal septum, we present the second described case of a lateral nasal cavity wall REAH in a 68-year-old male with a 2-year history of progressive left nasal obstruction. Clinical and radiological assessment predicted malignancy; however, histopathology identified a benign pathology. He was subsequently treated with narrow local excision under general anaesthetic with no evidence of recurrence at post-operative intervals.

## INTRODUCTION

Hamartomas are predominately benign tumours [1, 2], a local malformation of multiple aberrant cell types, rather than a single mutated cell proliferation [1, 3]. Respiratory epithelial adenomatoid hamartoma (REAH) arises from Schneiderian respiratory epithelium (pseudostratified respiratory epithelium), the surface epithelium within nasal cavity and sinonasal space [2, 4, 5].

**Figure 1:**
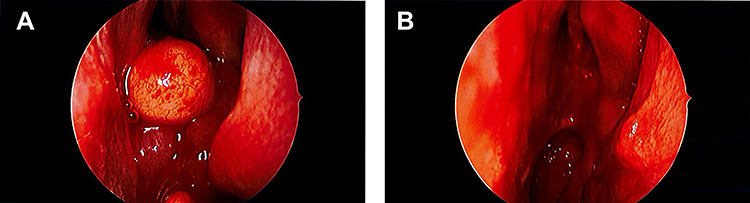
Intraoperative endoscopic images. (**A**) Large, spherical, well-circumscribed polypoidal tumour in the left nasal passage; (**B**) post-operative image showing complete excision of the lesion.

**Figure 2:**
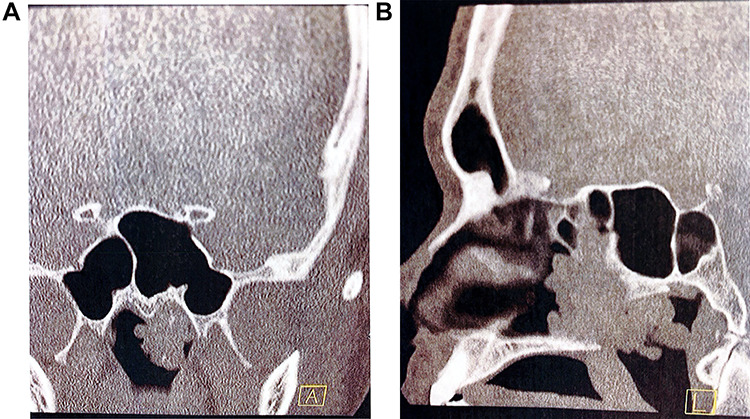
Pre-operative computed tomography of the nasal passage and sinuses. (**A**) Coronal CT sinuses (bone algorithm) demonstrating left nasopharyngeal space ‘polypoid’ type opacification, with evidence of intralesional calcification; (**B**) sagittal CT sinuses (bone algorithm) demonstrating a large polypoidal type opacification obstructing the posterior nasal choanae.

**Figure 3:**
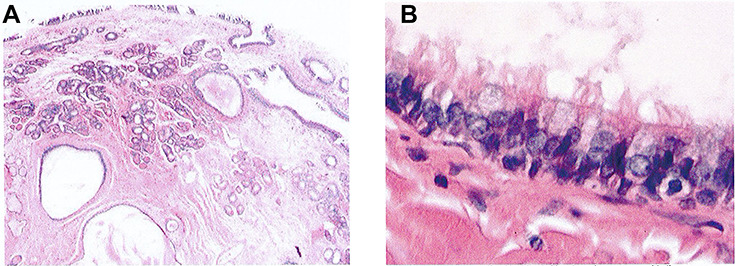
(**A**) Haematoxylin and eosin staining of lesion demonstrating cystic and glandular structures within a fibrous stroma (light microscopy, ×4); (**B**) haematoxylin and eosin staining of lesion demonstrating cysts lined by ciliated respiratory type epithelium (light microscopy, ×40).

During clinical and radiological assessment, REAH can masquerade as sinonasal carcinoma [2]. Surgical intervention and adjuvant therapies for sinonasal carcinoma (i.e. squamous cell, adenocarcinoma, adenoid cystic carcinoma, mucosal melanoma) are associated with significant morbidity [1, 2]; consequently, accurate histopathological assessment of REAH is important [4].

## CASE REPORT

A 68-year-old gentleman presented with a 2-year history of progressive left nasal obstruction. There was no associated dysgeusia or hyposomnia or anosmia. He was a lifelong non-smoker and was not known to have allergies to medications or an atopic type of rhinosinusitis. He did not have any other significant medical or surgical history and no genetic history of nasal pathologies.

On examination, he had severely reduced left nasal airflow. Anterior rhinoscopy revealed a large fleshy tumour in the left nasal cavity, which was not papillomatous or vascular (Fig. 1). Nasendoscopic examination showed a large tumour of solid and polypoidal components. It occluded the posterior nasal choanae, filling the inferior aspect of the nose. Visualized from the right nostril, the tumour prolapsed into the postnasal space. There was no contact bleeding. The right nasal passage appeared normal. The nasopharyngeal vault and the remainder of the upper airway appeared normal. He did not have otitis media with effusion in the left ear and there was no palpable cervical lymphadenopathy. Pre-operative imaging was ordered to determine the extent of the lesion (Fig. 2).

Treatment was local excision under general anaesthetic. The tumour was attached to the lateral wall of the nose at the level of the middle turbinate and was removed in its entirety excision. There was minimal bleeding and the nasal passage looked otherwise normal. Middle meatal antrostomy revealed scant mucus in the antrum, but the mucosa appeared normal. The perioperative course was uneventful, and the patient was discharged home on the sameday.

### Histopathology

#### Macroscopic

The resected mass was a grey polypoidal tissue with spicules of bone measuring 36 × 33 × 18 mm.

#### Microscopic

Histological examination revealed the mass was lined with ciliated respiratory epithelium, which invaginated into the core of the polyp, forming glandular and cystic structures. There was focal calcification within the cysts. There was a component of bland sero-mucinous glands in lobular arrangements. The epithelial elements were encased in a hyalinized fibrous stroma. No features of atypia or malignancy were visualized (Fig. 3).

#### Follow up

On follow up, he was asymptomatic and had normal nasal airflow, taste and smell.

## DISCUSSION

REAH is a sinonasal tumour characterized by glandular proliferation of the surface epithelium of the nasal cavity and paranasal sinuses [6]. Weinig and Heffner [4] first described it in an analysis of 31 cases.

REAH has the capacity to masquerade as malignancy; consequently, diagnosis is imperative to inhibit aggressive therapy. It is an important differential diagnosis because it can present similarly to other sinonasal lesions, such as inflammatory polyps, inverted papilloma, well-differentiated adenocarcinoma, squamous cell carcinoma, olfactory neuroblastoma and lymphomas [2, 5]. If REAH is misidentified as a more sinister lesion, it can result in over aggressive and/or deforming treatment for a patient [4].

This condition is most common in males aged 30–90 years [1]. Patients present with non-specific symptoms, such as nasal obstruction, epistaxis, hyposomia, chronic sinusitis, deviated septum and frontal headache [5].

Previous reports have identified REAH arising from the nasal septum (especially the posterior aspect); however, we describe the first case arising from the posterior nasal wall [4, 5, 7].

REAH is a complex diagnosis that often cannot be based on clinical findings or radiological evidence alone. REAH most commonly presents as either as a solitary lesion or in association with nasal polyposis, the latter being far more common [3].

Radiologically, the most common finding on a non-contrast-enhanced computed tomography (CT) sinus (bone algorithm) is opacification of sinuses whose ostea are blocked by a posterior nasal cavity polypoid lesion (namely, sphenoid and posterior ethmoid air cells) [1]. Vira *et al*. [8] retrospectively reviewed the perioperative CT scans of 35 patients with REAH and concluded that there were no distinguishing features that could differentiate it from any other sinonasal lesion. However, a later study found that a widened olfactory cleft, greater than 10 mm on CT imaging, is a characteristic feature of REAH [9]. The olfactory cleft is the distance between the both turbinal ethmoid walls and were significantly increased in patients with REAH in axial (12.2 mm) and coronal (12.1 mm) planes, when compared with patients with nasal polyposis (5.6 and 5.4 mm) and patients with no disease (4.5 and 4.2 mm) [10].

Ultimately, a diagnosis of REAH requires histopathological confirmation in order to definitively differentiate it from other lesions.

Grossly, REAH has a polypoid appearance, with fleshy or firm, yellow–white masses of various sizes [1].

Rom *et al*. [6] analysed the histopathology of nine samples and found that the tissue showed prominent glandular proliferation, with small- to medium-sized round to oval glands that are widely separated by stromal tissues. These glands are lined by multilayered ciliated respiratory epithelium with goblet cells and have a thick, eosinophilic basement membrane [2]. They do not present with atypia or metaplastic change [8].

With correct identification of REAH, it can be treated with simple excision, compared with sinister sinonasal lesions, which may require aggressive surgical debridement and adjuvant therapies [1]. With appropriate excision, recurrence is extremely rare [7].

## CONFLICT OF INTEREST STATEMENT

None declared.

## FUNDING

None.

## References

[1] Fitzhugh VA, Mirani N. Respiratory epithelial adenomatoid hamartoma: a review. Head Neck Pathol 2008;2:203–8. 20614315 10.1007/s12105-008-0064-3 PMC2807563

[2] Endo R, Matsuda H, Takahashi M, Masamichi H, Inaba H, Tsukuda M. Respiratory epithelial adenomatoid hamartoma in the nasal cavity. Acta Otolaryngol 2002;122:398–400. 12125996 10.1080/00016480260000085

[3] Gauchotte G, Marie B, Gallet P, Nguyen DT, Grandhaye M, Jankowski R, et al. Respiratory epithelial adenomatoid hamartoma: a poorly recognised entity with mast cell recruitment and frequently associated with nasal polyposis. Am J Surg Pathol 2013;37:1678–85. 24121171 10.1097/PAS.0000000000000092

[4] Wenig BM, Heffner CDK. Respiratory epithelial adenomatoid hamartomas of the sinonasal tract and nasopharynx: a clinicopathologic study of 31 cases. Ann Otol Rhinol Laryngol 1995;104:639–45. 7639474 10.1177/000348949510400809

[5] Delbrouck C, Fernandez Aguilar S, Choufani G, Hassid S. Respiratory epithelial adenomatoid hamartoma associated with nasal polyposis. Am J Otolaryngol 2004;25:282–4. 15239039 10.1016/j.amjoto.2004.02.005

[6] Rom D, Lee M, Chandraratnam E, Chin R, Sritharan N. Respiratory epithelial adenomatoid hamartoma: an important differential of sinonasal masses. Cureus 2018;10:e2495. 29922536 10.7759/cureus.2495 PMC6003788

[7] Villarreal IM, Pinilla M, Salas I, Garcia Y, López-Cortijo C. Respiratory epithelial adenomatoid hamartoma: a very rare entity originating from the lateral nasal wall. Eur Ann Otorhinolaryngol Head Neck Dis 2015;132:369–70. 26338515 10.1016/j.anorl.2015.08.003

[8] Vira D, Bhuta SF-W, Wang MB. Respiratory epithelial adenomatoid hamartomas. Laryngoscope 2011;121:2706–9. 22006652 10.1002/lary.22399

[9] Safi C, Li C, Tabaee A, Ramakrishna R, Riley CA. Outcomes and imaging findings of respiratory epithelial adenomatoid hamartoma: a systematic review. Int Forum Allergy Rhinol 2019;9:674–80. 30657648 10.1002/alr.22298

[10] Lima NB, Jankowski R, Georgel T, Grignon B, Guillemin F, Vignaud JM. Respiratory adenomatoid hamartoma must be suspected on CT-scan enlargement of the olfactory clefts. 2006;44:264–9. 17216743

